# Exploring the sub-Neptune frontier with JWST

**DOI:** 10.1073/pnas.2416194122

**Published:** 2025-09-22

**Authors:** Nikku Madhusudhan, Måns Holmberg, Savvas Constantinou, Gregory J. Cooke

**Affiliations:** ^a^Institute of Astronomy, University of Cambridge, Cambridge CB3 0HA, United Kingdom; ^b^Space Telescope Science Institute, Baltimore, MD 21218

**Keywords:** exoplanets, sub-Neptunes, exoplanet atmospheres, interiors, habitability

## Abstract

Sub-Neptune planets, with sizes and masses between those of Earth and Neptune, dominate the exoplanet population. Sub-Neptunes are expected to be the most diverse family of the exoplanet population, potentially including rocky gas dwarfs, water worlds, and mini-Neptunes, with a wide range of atmospheric, surface, and interior conditions. With no analogue in the solar system, these planets open fundamental questions in planetary processes, origins, and habitability, and present avenues in the search for life elsewhere. Atmospheric observations with the James Webb Space Telescope (JWST) are enabling unprecedented characterization of sub-Neptunes, starting with the first detections of carbon-bearing molecules in the habitable zone sub-Neptune K2-18 b. We survey the present landscape of JWST observations and atmospheric inferences of sub-Neptunes, which in turn provide key insights into their atmospheric processes, internal structures, surface conditions, formation pathways, and potential habitability. The atmospheric abundance constraints reveal evidence of chemical disequilibria, and insights into the planetary mass–metallicity relation in the sub-Neptune regime. Similarly, for sub-Neptunes with H_2_O-rich interiors, increasing atmospheric H_2_O abundances with the equilibrium temperature may indicate the existence of a critical temperature for transition from H_2_ dominated atmospheres with tropospheric cold traps to those with steamy atmospheres. The chemical abundances also provide initial evidence for diverse planet types, from potentially habitable hycean worlds to steam worlds with supercritical water layers. These planet types serve as benchmarks for an emerging taxonomy of volatile-rich sub-Neptunes as a function of their equilibrium temperature and atmospheric extent, heralding a new era of chemical classification of low-mass exoplanets with JWST.

## Significance

Sub-Neptune planets dominate the exoplanet population but have no analogues in the solar system. Sized between Earth and Neptune, the nature of such planets remains uncertain, spanning rocky gas dwarfs, mini-Neptunes, and water worlds, with some potentially capable of harboring habitable conditions. Sub-Neptunes are therefore a central focus in the study of planetary processes in low-mass exoplanets with many open questions. Pioneering JWST observations have led to detections of prominent molecules in several sub-Neptune atmospheres. The chemical abundances are providing initial insights into compositional trends, with important implications for their atmospheric diversity, internal structures, formation mechanisms, and habitability. These results set the stage for a generalized classification of volatile-rich sub-Neptunes and offer an early panoramic view of this exotic frontier.

We are at the beginning of a new era in exoplanetary science. Exoplanet demographics reveal that planets between Earth and Neptune in size dominate the known exoplanet population. Sub-Neptunes typically refer to planets with radii between ∼1.5 and 4 R_⊕_, smaller than Neptune but larger than rocky planets with thin atmospheres (1–3). Sub-Neptunes are expected to span a wide range of planet types (4–7), including planets with rocky interiors and thick H_2_-rich envelopes (gas dwarfs), smaller versions of Neptune (mini-Neptunes) with volatile-rich interiors and H_2_-rich envelopes, and water worlds with H_2_O-dominated interiors and varied atmospheric compositions. The water worlds can include steam worlds with H_2_O-dominated atmospheres, ocean worlds with terrestrial-like atmospheres (8) and hycean worlds with H_2_-rich atmospheres (9). The central enigma underlying the sub-Neptune frontier is the lack of any such planet in the solar system which can serve as a reliable archetype. The abundance of sub-Neptunes has opened an unprecedented and uncharted discovery space regarding their formation mechanisms, interior and surface processes, atmospheric diversity, and potential for habitability.

A defining feature of the sub-Neptune population is the existence of a “Radius Valley” (1), a bimodal distribution in planetary radii centered around 1.8 R_⊕_ with peaks near 1.4 R_⊕_ and 2.4 R_⊕_. The origin of this distribution is one of the most fundamental open questions in exoplanet science with important implications for the formation and nature of low-mass planets (3, 10). Two contesting hypotheses have been put forward to explain the distribution with different formation and evolutionary pathways. In one scenario, the two peaks in the distribution are both populated by rocky planets, but with the larger population retaining their primordial H_2_-rich envelopes whereas the smaller ones having lost their envelopes through photoevaporation or core powered mass loss (11, 12). In the second scenario, planets in the smaller peak are still rocky planets without significant envelopes but those in the larger peak are primarily volatile-rich planets with a large inventory (up to tens of percent) of water in the interiors along with H_2_-rich envelopes (7, 13–15), owing to volatile accretion and migration. Each of the scenarios have implications for the internal structures and atmospheric compositions. Bulk parameters, such as mass, radius, and density, are alone not sufficient to differentiate between the different possibilities for their internal structures (3–5). Therefore, atmospheric observations of sub-Neptunes are critical to provide deeper insights into their compositions and origins.

The sub-Neptune regime also represents a missing link in our understanding of planetary processes. In the solar system, there is a marked difference between the atmospheres of terrestrial planets (e.g., Earth, Venus, and Mars) and those of ice giants (Neptune and Uranus). While the former host thin secondary atmospheres dominated by heavy molecules like N_2_ or CO_2_, the latter have deep H_2_-dominated primary atmospheres with only trace amounts of heavier molecules like CH_4_ and NH_3_ (16). As sub-Neptunes span a continuum in bulk properties between terrestrial planets and ice giants, their atmospheres can be expected to span a diverse range of physical and chemical processes (e.g., refs. 17–22), including new avenues for planetary habitability (9). Therefore, sub-Neptune planets present a fortuitous opportunity to study a continuum of planetary processes in the low-mass regime as well as to discover key transitions and new processes in this uncharted territory.

Atmospheric characterization of sub-Neptune planets has remained a formidable barrier in the pre-JWST era. Starting with the early observations with the Hubble Space Telescope (HST) of a featureless transmission spectrum of the sub-Neptune GJ 1214 b (31) a decade ago, obtained with ∼90 h of HST time, substantial efforts have been dedicated to such planets. Some successes with HST were seen in the inferences of atmospheric features in the 1.1 to 1.7 μm range, which overlaps with the JWST NIRISS instrument, for a few temperate sub-Neptunes orbiting bright M dwarfs such as K2-18 b (32, 33), TOI-270 d (26), and GJ 9827 d (34), also with substantial HST time in some cases, e.g., over 50 h for K2-18 b. While the observed features for K2-18 b were initially attributed to H_2_O absorption (6, 32, 33) with notable upper limits on CH_4_ (6), significant degeneracies remained with other potential contributions, e.g., with CH_4_ (35, 36) and stellar contamination (37). Furthermore, the possibility of clouds/hazes attenuating the spectral features in transmission spectroscopy was also considered to be a significant challenge for characterizing temperate atmospheres (e.g., refs. 31 and 38). More recently, it has also been suggested that the prevalence of clouds/hazes may not have a linear dependence on temperature, with higher propensity for clouds/hazes in the ∼500 to 800 K range and clearer atmospheres for more temperate planets (39, 40). HST continues to make important contributions toward understanding sub-Neptune atmospheres, especially with regard to atmospheric escape (e.g., ref. 41). However, the limited spectral range and sensitivity available in the pre-JWST era rendered detailed characterization of sub-Neptune atmospheres challenging at a population level.

The advent of JWST has transformed this frontier overnight. With only 14 h of observations in the first year of JWST, a near-infrared (0.8 to 5.2 μm) transmission spectrum of K2-18 b (42) led to the first detections of carbon-bearing molecules in a temperate exoplanet, resolving the longstanding missing methane problem and the degeneracy between CH_4_ and H_2_O. It also provided evidence for chemical disequilibrium in a habitable zone^*^ exoplanet, and important constraints on the possible internal structure, surface conditions, and potential habitability. In subsequent observations, chemical constraints have also been reported for several other sub-Neptunes (e.g., refs. 45–51). These observations have wide ranging implications for sub-Neptune atmospheres, interiors, formation pathways, and habitability. These developments have made the study of sub-Neptunes one of the most dynamic frontiers in exoplanetary science. In this work, we present a panoramic glimpse into this exotic landscape as observed with JWST.

In what follows, we present an overview of current observations of sub-Neptune planets with JWST and the resulting inferences. We subsequently discuss constraints on a range of physical and chemical processes in sub-Neptune atmospheres. We then consider constraints on their possible internal structures and surface conditions which in turn motivate a generalized classification of volatile-rich sub-Neptunes. We finally discuss the prospects for habitable conditions on temperate sub-Neptunes.

## JWST Observations

JWST is revolutionizing atmospheric spectroscopy of sub-Neptunes. Here, we discuss atmospheric detections reported for promising targets, and early lessons on the challenges and promises with JWST.

### Sub-Neptune Observations with JWST.

**Fig. 1.**
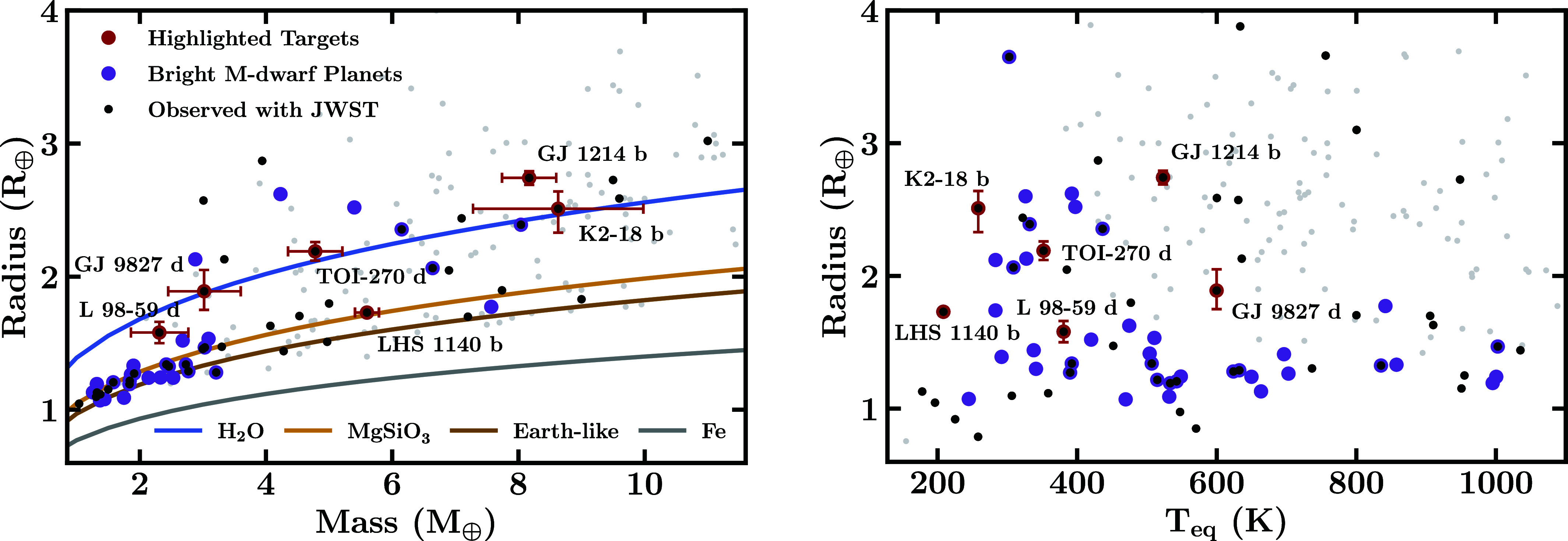
Bulk properties of promising sub-Neptunes. The black points represent targets observed with JWST, while purple points indicate sub-Neptunes orbiting bright M-dwarfs with $J_{\mathrm{mag}}<10$ and $M_{*}<0.5\;M_{⊙}$, favorable for atmospheric characterization. Gray points denote other exoplanets with precise mass and radius measurements. Six targets of interest with recent JWST observations are shown in red along with the uncertainties on their masses and radii: K2-18 b (23, 24), TOI-270 d (25, 26), GJ 9827 d (27), GJ 1214 b (28), LHS 1140 b (29), and L 98-59 d (30). For the equilibrium temperatures, $T_{\mathrm{eq}}$, we assume a Bond albedo of $A_{B}=0.3$ and full redistribution. The mass–radius curves in the left panel are from ref. 6 and the planet properties are obtained from the NASA Exoplanet Archive.

**Fig. 2.**
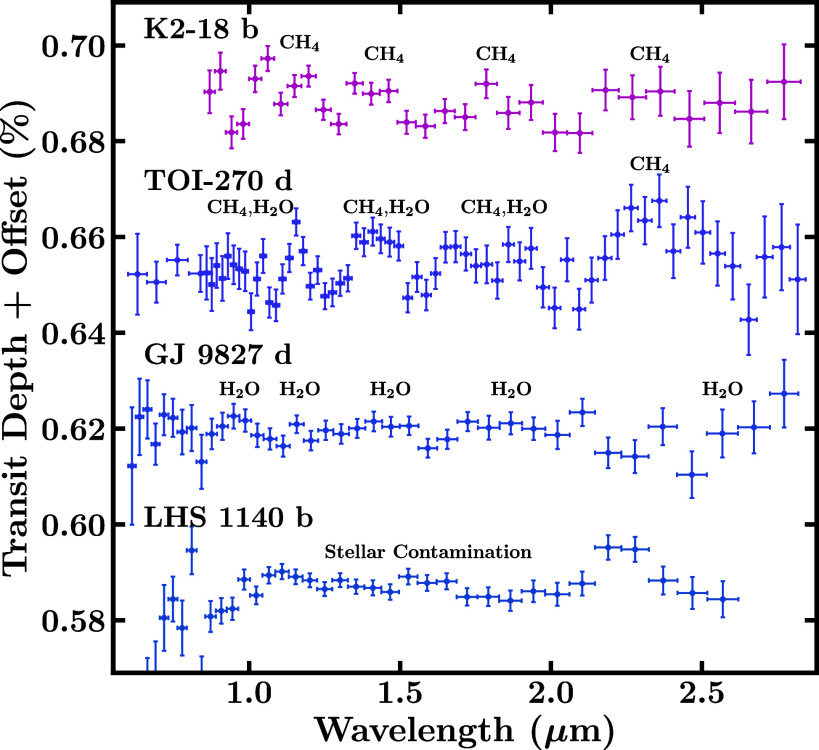
JWST transmission spectra of four sub-Neptunes in the 0.6 to 2.8 μm wavelength range obtained with NIRISS SOSS. These spectra of K2-18 b, TOI-270 d, GJ 9827 d and LHS 1140 b are obtained from refs. 42, 46, 47, and 55, respectively. For the transmission spectra of GJ 9827 d and LHS 1140 b, we binned every four spectral points for visual clarity. The spectral features of prominent molecules as inferred in the respective works are labeled.

**Fig. 3.**
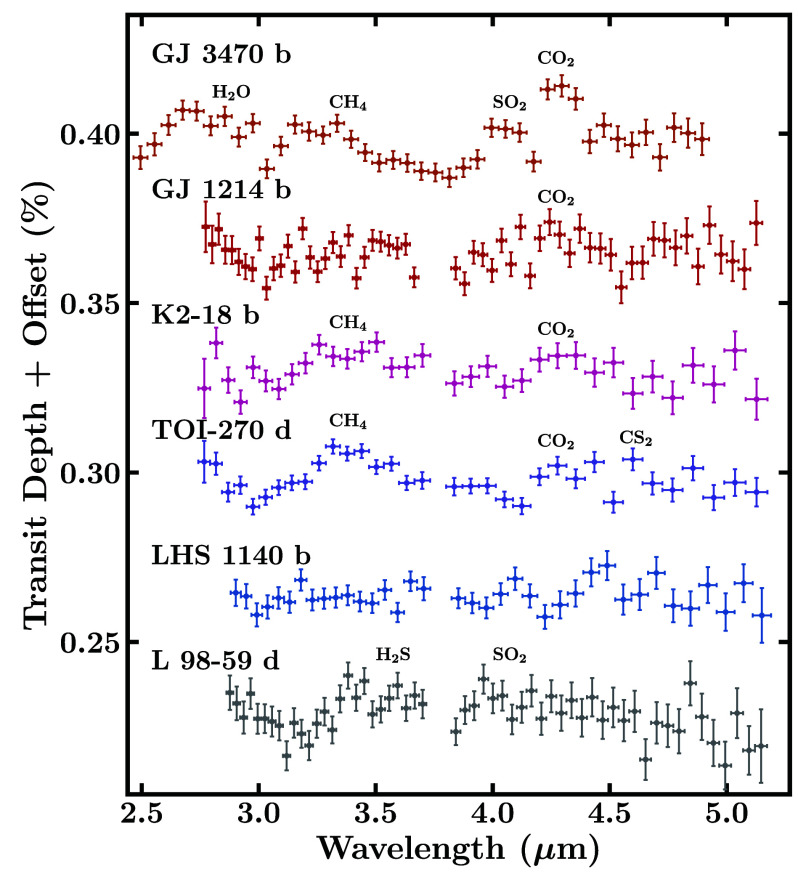
JWST transmission spectra of six sub-Neptunes in the 2.5 to 5.2 μm wavelength range obtained with NIRSpec G395H or NIRCam F322W2/F444W. The spectra of GJ 3470 b, GJ 1214 b, K2-18 b, TOI-270 d, LHS 1140 b, and L 98-59 d are obtained from refs. 53, 54, 42, 45, 48, and 50, respectively. In the case of GJ 3470 b, we binned every four spectral points for visual clarity. The spectral features of prominent molecules as inferred in the respective works are labeled. The LHS 1140 b spectrum has been inferred as showing no prominent spectral features (48), instead favoring a high-mean-molecular-weight atmosphere.

A diverse set of sub-Neptunes have been observed with JWST. Fig. 1 shows a population of sub-Neptunes as a function of mass, radius, and equilibrium temperature (*T*_eq_), highlighting key planets with JWST observations and promising targets for atmospheric characterization. JWST detections of prominent molecules have been reported in the atmospheres of several promising sub-Neptunes, starting with the habitable zone sub-Neptune K2-18 b (42), followed by somewhat warmer planets TOI-270 d (45, 46) and GJ 9827 d (47). These observations utilized a combination of NIRISS SOSS and/or NIRSpec G395H. Additionally, robust detections have also been reported for a warm exo-Neptune GJ 3470 b (52), using NIRCam F322W2 and F444W (53), which serves as an end-member case (being Neptune-sized), for the sub-Neptune regime. Moreover, the sub-Neptunes GJ 1214 b, LHS 1140 b, and L 98-59 d (TOI-175 d) have also been observed using transmission spectroscopy with JWST (48, 50, 54, 55), with some evidence for atmospheric features. Figs. 2 and 3 illustrate the recent transmission spectra obtained for these targets.

The coolest of these targets, and the first to be observed with JWST, is K2-18 b, a habitable zone sub-Neptune with an equilibrium temperature of $T_{\mathrm{eq}}=258$ K (assuming $A_{B}=0.3$ and full redistribution). The JWST transmission spectrum of K2-18 b revealed prominent spectral features of CH_4_ and CO_2_ (42)—marking the first detection of carbon-bearing molecules in a habitable zone exoplanet. With a higher temperature of $T_{\mathrm{eq}}=352$ K, TOI-270 d is another temperate sub-Neptune observed with JWST. The transmission spectrum of TOI-270 d led to robust detections of CH_4_ and CO_2_, with further evidence for CS_2_ and a potential inference of H_2_O (45, 46). Similar to K2-18 b, NH_3_ and CO were not detected in the atmosphere of TOI-270 d. However, the spectrum of TOI-270 d did not provide strong evidence for clouds/hazes, in contrast to K2-18 b, which showed a ∼3σ preference for the presence of clouds/hazes. Additionally, the transmission spectrum of GJ 9827 d, a hotter sub-Neptune with a temperature of *T*_eq_ = 600 K, showed spectral features of mainly H_2_O by combining two visits with NIRISS SOSS (47), although with less prominent spectral signatures compared to K2-18 b and TOI-270 d. Finally, the JWST spectrum of exo-Neptune GJ 3470 b, with an equilibrium temperature of *T*_eq_ = 634 K, exhibits prominent features of H_2_O, CH_4_, CO_2_, and SO_2_ (53).

Besides these four targets, several other sub-Neptune exoplanets have been observed with JWST, as mentioned above, such as LHS 1140 b (NIRISS SOSS, NIRSpec G235H/G395H; 48, 55), GJ 1214 b (MIRI LRS, NIRSpec G395H; 49, 54), L 98-59 d (NIRSpec G395H; 50, 56) and 55 Cancri e (in emission, using MIRI LRS and NIRCam F444W; 51, 57). Refs. 48 and 55 found evidence against a H_2_-rich atmosphere on the habitable zone sub-Neptune LHS 1140 b, with the data favoring a high mean molecular weight (MMW) atmosphere with additional contribution from unocculted stellar heterogeneities at shorter wavelengths. For GJ 1214 b, a warm sub-Neptune found to host an atmosphere dominated by clouds (31, 49), CO_2_, and CH_4_ were tentatively inferred (54, 58). However, the robustness of these detections is hindered by the possibility of clouds/hazes. Similarly, atmospheric spectroscopy of L 98-59 d, a sub-Neptune slightly cooler than GJ 1214 b has revealed tentative indications of sulfur-bearing species H_2_S and SO_2_ (50, 56), with these molecules potentially resulting from tidal-driven volcanism (59). Last, 55 Cancri e, a strongly irradiated sub-Neptune, with *T*_eq_ ∼ 2,000 K, was found to potentially host an atmosphere, with indications of it primarily being composed of CO and CO_2_ (51). Further observations of these targets can confirm the above findings which for now remain tentative. Moreover, the above list will undoubtedly expand as more sub-Neptunes across the temperature range are presently under observation with JWST. Other JWST studies of sub-Neptunes reveal seemingly featureless spectra (e.g., refs. 60 and 61). Overall, these results highlight the diversity of sub-Neptune exoplanets, emerging trends, and early lessons.

### Early Lessons.

JWST Cycles 1 to 3 have dedicated around 800 h to transit spectroscopic observations of sub-Neptunes across more than 40 unique targets. However, only a subset of observed exoplanets has resulted in robust atmospheric detections, as described above. Specifically, published JWST observations of K2-18 b, TOI-270 d, GJ 9827 d, and GJ 3470 b amount to only about 110 h so far. These observations provide important insights for successful atmospheric characterization of sub-Neptunes using JWST.

**Fig. 4.**
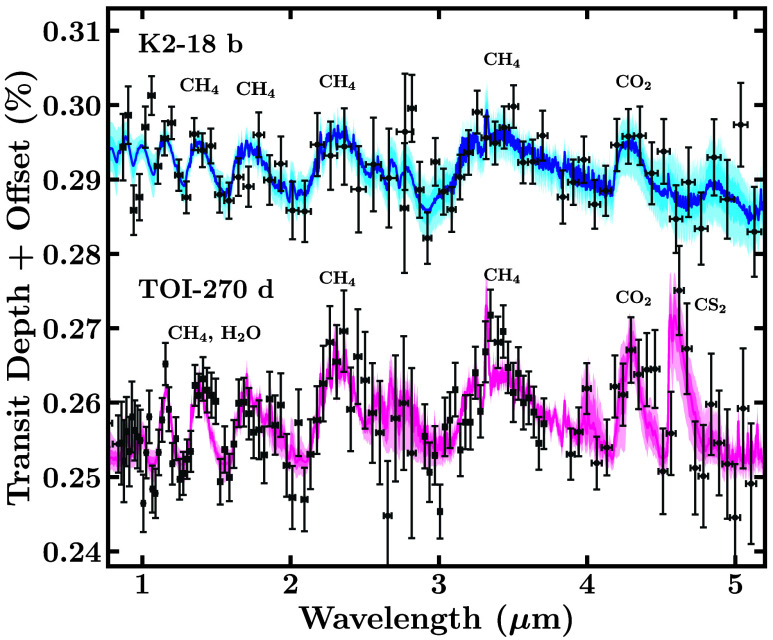
JWST transmission spectra of K2-18 b and TOI-270 d, observed with NIRISS SOSS and NIRSpec G395H. The K2-18 b and TOI-270 d data are obtained from refs. 42 and 46, respectively, and shown in gray squares with error bars. The colored contours show nominal model fits to the data, with the dark curves denoting the median retrieved model spectra, while the lighter contours denote the 1*σ* and 2*σ* intervals. Molecules with prominent spectral features are labeled.

One of the early identified challenges for atmospheric characterization of small exoplanets is the presence of stellar heterogeneities and flares. These factors can contaminate the observed transmission spectrum, complicating the analysis. This issue has proven particularly significant for JWST observations of exoplanets orbiting late M-dwarfs, as reported for TRAPPIST-1 (62, 63) and other systems (e.g., ref. 64). Successful JWST observations of mid-M dwarfs, such as K2-18 and TOI-270 (Fig. 4), clearly demonstrate that low stellar activity is essential for mitigating these effects.

Additionally, the temperature of the planet plays a crucial role in its observability, all other factors remaining the same. For planets with a large H_2_O content, high temperatures can lead to a mixed H_2_–H_2_O envelope with high MMW (46, 65), as described in the *Sub-Neptune Interiors and Surface Conditions* section, reducing the overall amplitude of the spectral feature. At the same time, both observations and theoretical studies also seem to indicate that planets with $T_{\mathrm{eq}}$ below ∼500 K are less likely to be affected by clouds/hazes attenuating transmission spectra (39, 40, 42, 45). Based on the above findings, temperate sub-Neptunes with $T_{\mathrm{eq}}≲500$ K orbiting relatively quiet stars appear ideal for atmospheric characterization. For optimal results, it is also vital that the host star is bright enough, e.g., J<10 (9). These considerations make target selection particularly important for reliable atmospheric characterization of sub-Neptunes.

Moreover, several systematics have been identified to be important for transit observations of small planets with JWST. Notable among these are offsets or broadband variations possible in the transmission spectrum across different instruments, detectors, and visits (42, 47, 64). Key factors that may contribute to such variations include treatment of limb darkening, light-curve detrending, stellar variability and possible instrumental effects (42, 45, 55, 60). Some of these effects, such as offsets or stellar heterogeneities, can be considered in the atmospheric retrievals (42, 60) while also being potentially degenerate with some of the atmospheric model parameters. Additionally, correlated noise is another potential concern affecting some sub-Neptune observations (e.g., refs. 57, 61, and 66). This requires careful attention to the treatment of noise properties, with multiple transits preferred for robustness (60). Overall, it is crucial to thoroughly investigate the effects of various data analysis assumptions and approaches to ensure robust inference and interpretation.

## Atmospheric Retrievals

The JWST spectra discussed above are providing the first detailed constraints on the atmospheric properties of several sub-Neptunes using atmospheric retrieval methods (67). As most of the chemical detections for sub-Neptunes to date have been made using transmission spectroscopy, the retrieved properties pertain to the observable atmosphere at the day-night terminator region. The retrieved properties include volume mixing ratios of prominent molecular species along with constraints on the temperature structure, the presence of clouds/hazes, and the effect of stellar contamination.

**Fig. 5.**
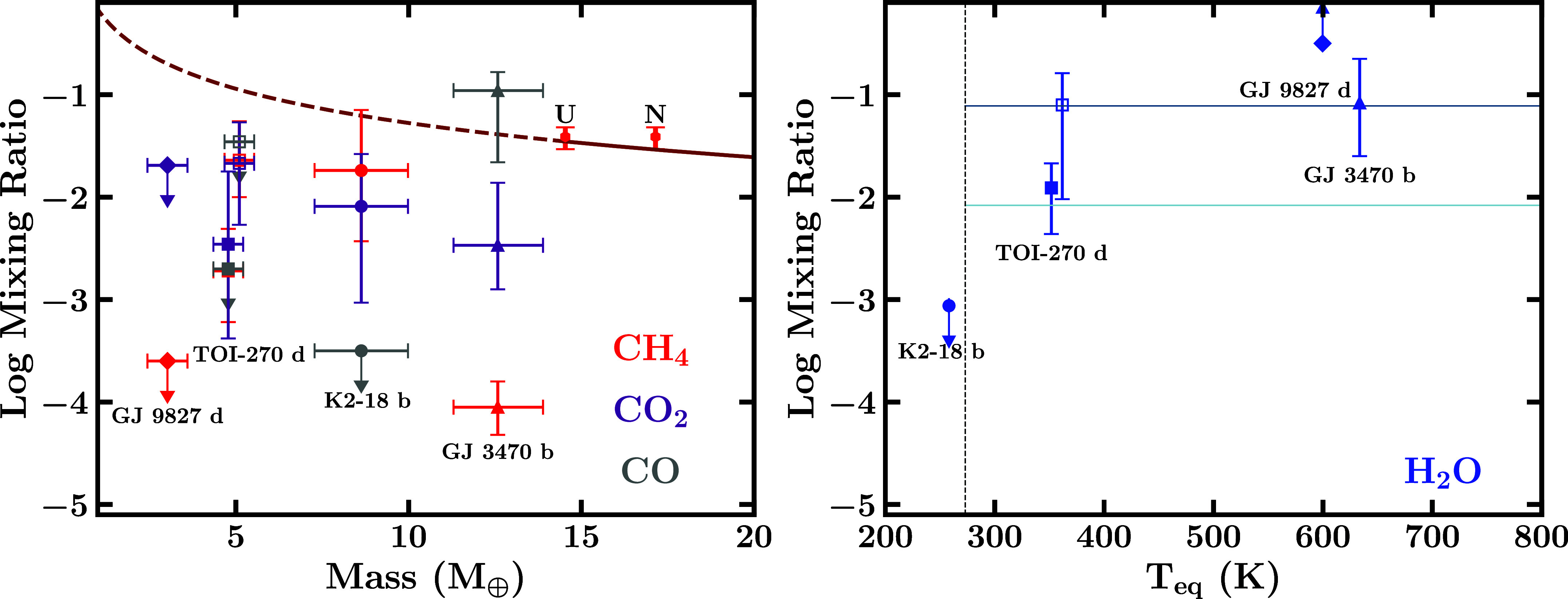
Composition trends in sub-Neptune atmospheres. Points with errorbars denote the median and 1σ uncertainties in retrieved abundances of prominent molecules, while arrows denote a 2σ upper or lower limit. The constraints are shown for four planets: K2-18 b (42), TOI-270 d (45, 46), GJ 9827 d (47) and GJ 3470 b (53). For TOI-270 d we show abundances reported in two independent works, denoted by filled squares (45) and open squares (46). *Left*: CH_4_ (red) CO_2_ (purple) and CO (gray) constraints against planetary mass, along with CH_4_ constraints for Uranus and Neptune (16). Also shown is a fitted mass–metallicity trend for solar system giant planets (including Saturn and Jupiter) as a solid brown line, and its extrapolation to the sub-Neptune regime as a dashed brown line. *Right*: H_2_O mixing ratio constraints for the same four targets against their equilibrium temperatures assuming a 0.3 Bond albedo. Here, the mixing ratio of a molecule is the ratio of its number density relative to the total number density. Light and dark blue lines denote the gaseous H_2_O volume mixing ratio for atmospheres with a 10× and 100× solar oxygen abundance, respectively, assuming all oxygen is in H_2_O. The vertical black dashed line indicates the 273 K condensation temperature for H_2_O under tropospheric conditions for illustrative purposes.

Here, we focus on the atmospheric constraints for three sub-Neptune targets for which high-confidence molecular detections have been reported with high-precision JWST transmission spectra. These include K2-18 b (42), TOI-270 d (45, 46) and GJ 9827 d (47). We also discuss the retrieved constraints for the exo-Neptune GJ 3470 b (53) for a comparative assessment between the sub-Neptune targets and a hot ice-giant analogue in size. The retrieved chemical abundances of prominent molecules from published works for these targets are shown in Fig. 5.

### Atmospheric Constraints with JWST Observations.

The high-precision JWST spectra have led to precise abundance constraints for several prominent molecules. For K2-18 b, the observations led to robust detections of prominent carbon-bearing molecules methane (CH_4_) and carbon dioxide (CO_2_) in a H_2_-rich atmosphere with retrieved abundances of log(${\text{CH}}_{4})=-1.74_{-0.69}^{+0.59}$ and log(CO_2_) = $-2.09_{-0.94}^{+0.51}$ (one-offset case; 42). The observations did not show evidence for other prominent molecules such as H_2_O, NH_3_, and CO, and provided 2σ upper limits of −3.06, −4.51, and −3.50, respectively, on their log mixing ratios. The retrievals obtained nominal constraints on the presence of clouds/hazes, which were preferred at a 3*σ* level. The photospheric (10 mbar) temperature at the terminator was constrained to be $242_{-57}^{+79}$ K, consistent with the nondetection of H_2_O which would be condensed out.

JWST observations of the temperate sub-Neptune, TOI-270 d, led to similar atmospheric detections and abundance constraints of carbon-bearing molecules as for K2-18 b, from two recent independent studies (45, 46). As discussed above, both works reported confident detections of CH_4_ and CO_2_, and potential inferences of CS_2_ and H_2_O. The abundance constraints reported by these works range between: log(CH_4_) = $-2.72_{-0.50}^{+0.41}$, log(CO_2_) = $-2.46_{-0.92}^{+0.71}$, log(H_2_O) =$-1.91_{-0.94}^{+0.57}$, log(CS_2_) = $-3.07_{-0.91}^{+0.74}$ (dual transit case; 45), and log(CH_4_) = $-1.64_{-0.36}^{+0.38}$, log(CO_2_) = $-1.67_{-0.60}^{+0.40}$, log(H_2_O) =$-1.10_{-0.92}^{+0.31}$, log(CS_2_) = $-3.44_{-0.67}^{+0.66}$ (46). Similarly to K2-18 b, no evidence was found for NH_3_ or CO in TOI-270 d.

JWST observations were also reported for a third, significantly warmer, sub-Neptune, GJ 9827 d (47). The spectrum showed strong evidence for a H_2_O-dominated atmosphere, with a 2σ lower limit of log(H_2_O) >−0.5, i.e., volume mixing ratio above 31.6%. The very high H_2_O abundance, unlike the temperate sub-Neptunes discussed above, leads to significantly smaller spectral amplitudes despite the higher temperature ($T_{\mathrm{eq}}=600$ K).

Finally, the warm exo-Neptune GJ 3470 b was recently observed with JWST NIRCam (53), leading to above 3σ detections of H_2_O, CH_4_, CO_2_, and SO_2_ in a H_2_-rich atmosphere (53). The abundances were reported to be log(H_2_O) = $-1.08_{-0.52}^{+0.43}$, log(CH_4_) = $-4.05_{-0.27}^{+0.25}$, log(CO_2_) = $-2.47_{-0.43}^{+0.61}$, and log(SO_2_) = $-3.57_{-0.25}^{+0.26}$. Additionally, ref. 53 also reported the abundance constraint of CO, albeit with a lower detection significance of 1.5σ, to be log(CO) = $-0.96_{-0.70}^{+0.18}$. This means that CO is potentially the most dominant carbon-bearing species in GJ 3470 b’s atmosphere, consistent with expectations from vertical mixing in the atmosphere.

As discussed in *JWST Observations*, nominal chemical signatures have also been inferred in some other sub-Neptunes with preliminary constraints on their abundances. For example, a tentative inference of CH_4_ and CO_2_ was reported for the warm sub-Neptune GJ 1214 b, with log(CH_4_) = $-2.25_{-0.45}^{+0.72}$ in a CO_2_-dominated atmosphere of $91_{-45}^{+8}$% by volume (54, 58). Given the low combined significance of CH_4_ and CO_2_, of 2 to 3σ, these studies suggest that more observations are required to detect both molecules independently and derive robust abundance estimates. Similarly, a sulfur-rich atmosphere was inferred for the sub-Neptune L 98-59 d, with a combination of H_2_S and SO_2_ reported at 2 to 4σ significance across multiple studies (50, 56). The abundances are constrained to log(H_2_S) = $-0.74_{-0.49}^{+0.14}$ by ref. 50 and log(H_2_S) = $-0.62_{-6.73}^{+0.61}$ by ref. 56. Additionally, ref. 56 infer log(SO_2_) = $-2.35_{-5.88}^{+2.05}$, while ref. 50 obtain a bimodal inference constraining log(SO_2_) to either above −1.0 or below −1.5.

Overall, atmospheric abundance constraints have been obtained for a small sample of promising sub-Neptunes to date with more observations for a larger target sample underway. These atmospheric constraints combined with self-consistent theoretical models can provide important insights into various atmospheric processes, interior and surface conditions, formation mechanisms, and potential for habitability as detailed below.

## Atmospheric Processes

The most direct inferences derived from retrieved atmospheric constraints are those of atmospheric processes. Such processes result in characteristic abundances of specific chemical species or groups of species relative to expectations from thermochemical equilibrium. Therefore, the atmospheric constraints can provide insights into chemical disequilibrium processes, temperature structures, clouds/hazes, atmospheric metallicity, and atmospheric extent. The atmospheric constraints at the population level can also enable key insights into planetary formation conditions and mechanisms, based on trends in atmospheric compositions with system properties including planet mass and irradiation.

### Chemical Disequilibrium and Atmospheric Extent.

All planetary atmospheres in the solar system are in chemical disequilibrium (68). Low temperature atmospheres are particularly susceptible to chemical, radiative, and dynamical processes which can drive the atmosphere out of thermochemical equilibrium (69, 70). For example, deep H_2_-rich atmospheres in chemical equilibrium are expected to be abundant in CH_4_ and NH_3_ as the dominant carriers of C and N, respectively, at low temperatures, and are replaced with CO and N_2_ at higher temperatures. At 1 bar, the CO–CH_4_ transition happens at ∼1,200 K and that for N_2_–NH_3_ at ∼700 K (69, 71, 72). However, vertical mixing can dredge up CO from the hotter lower atmosphere to the observable upper atmosphere even in temperate planets (19, 73). On the other hand, photochemical processes can result in destruction of CH_4_ and NH_3_ and enhancement of CO_2_ and HCN. In addition, the presence of a shallow surface underlying a thin H_2_-rich atmosphere can significantly affect the atmospheric composition by curtailing the thermochemical recycling from the lower atmosphere (18–20, 74). H_2_O is expected to be the dominant oxygen-bearing molecule in temperate H_2_-rich atmospheres but can be limited by condensation in a cool atmosphere or enhanced in the presence of a warm ocean surface or a metal-rich interior (e.g., refs. 46 and 74).

The retrieved atmospheric abundances of sub-Neptunes with JWST reveal diverse disequilibrium processes in their atmospheres and potential surface–atmosphere interactions. The first JWST detection of chemical disequilibrium in a temperate sub-Neptune was made for K2-18 b (42). The high retrieved abundances of CH_4_ and CO_2_, and nondetection of NH_3_ and CO simultaneously are incompatible with expectations from thermochemical equilibrium at the retrieved photospheric temperatures below 300 K. While the CH_4_ abundance alone is consistent with up to ∼150× solar metallicity in chemical equilibrium, the remaining abundances still need some form of chemical disequilibrium. However, chemical disequilibrium alone cannot explain the abundances if a deep H_2_-rich atmosphere is assumed (75, 76), necessitating the consideration of a thin H_2_-rich atmosphere over an ocean surface, as discussed further in the *Sub-Neptune Interiors and Surface Conditions* section. The similar abundance pattern observed for TOI-270 d (45, 46) presents a second instance of chemical disequilibrium in a temperate sub-Neptune with varied implications for the interior depending on the retrieved metallicity, as discussed below.

The chemical abundances of the exo-Neptune GJ 3470 serve as an important reference for interpreting sub-Neptune compositions (53). A limiting degeneracy in the interpretation of a sub-Neptune composition is whether the planet has a thin or deep H_2_-rich atmosphere, e.g., as in the case of K2-18 b discussed above. In the case of GJ 3470 b, the mass and radius of the planet are comparable to Uranus and Neptune, and, hence, require a deep H_2_-rich envelope in the interior. This breaks the degeneracy with regard to the atmospheric extent. The retrieved atmospheric abundance constraints of high H_2_O and CO_2_, low CH_4_, and high CO, albeit with marginal detection significance, are consistent with expectations of a warm and deep H_2_-rich atmosphere with vertical mixing (53). The nondetection of NH_3_ in this case is also consistent with the warm atmosphere.

### Clouds/Hazes and MMW.

In the pre-JWST era, it was expected that clouds/hazes would mute spectral features of temperate exoplanets, as exemplified by the featureless HST transmission spectrum of GJ 1214 b in the 1 to 1.7 μm range (31). Other studies have also discussed trends related to clouds/hazes in sub-Neptunes suggesting an intermediate temperature range of ∼500 to 700 K, where spectral attenuation due to clouds/hazes may be expected to be most significant (39, 40). As discussed above, JWST observations have successfully detected atmospheric features in the spectra of several low-mass exoplanets across a wide range of conditions, with temperatures ranging from $T_{\mathrm{eq}}≲300$ K for K2-18 b to $T_{\mathrm{eq}}∼600$ K for GJ 9827 d and GJ 3470 b. There have also been inferences of clouds/hazes in some sub-Neptunes, such as GJ 1214 b with $T_{\mathrm{eq}}\;∼\;$550 to 600 K (49, 54, 58) and K2-18 b (42), while still allowing for identification of significant spectral features. On the other hand, a strong scattering slope suggested for GJ 3470 b using HST (77) was found to be inconsistent with recent JWST spectra, which showed strong features of prominent molecules (53). It is generally the case, however, that more temperate sub-Neptunes, e.g., $T_{\text{eq}}$ below 400 K, are showing stronger spectral features compared to their warmer counterparts for comparable masses and radii as demonstrated for K2-18 b and TOI-270 d discussed above. More precise JWST observations of sub-Neptunes over a broader planetary temperature range would be instrumental for robustly constraining any underlying trends regarding the prevalence and nature of clouds/hazes in the sub-Neptune regime.

**Fig. 6.**
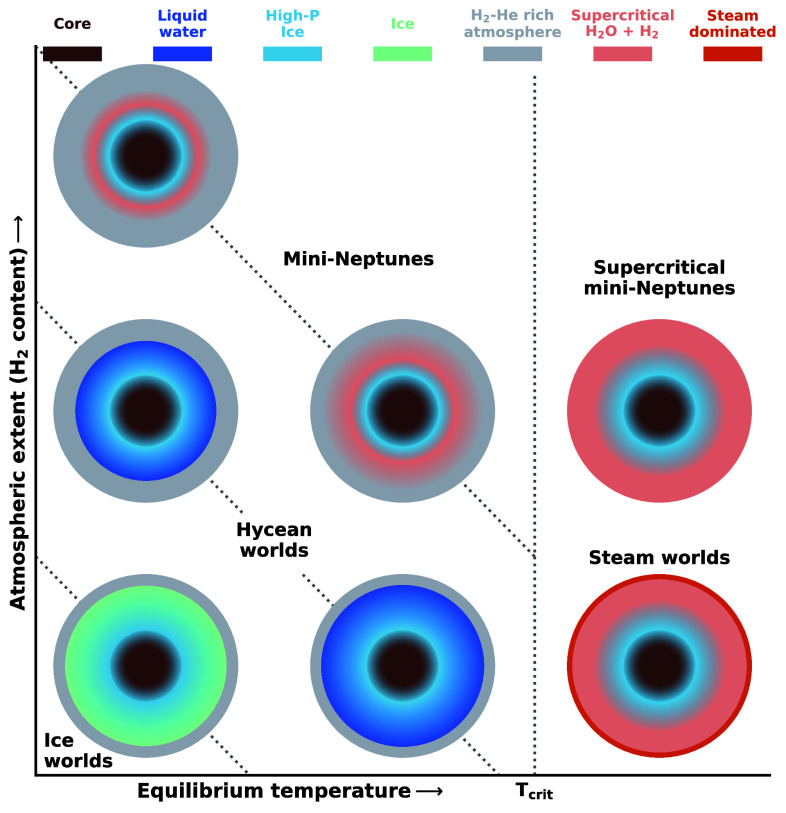
A classification scheme for volatile-rich sub-Neptunes. The schematic compares different types of sub-Neptunes with H_2_ and H_2_O dominated atmospheres as a function of the equilibrium temperature and H_2_ content. **Ice worlds**: Cold sub-Neptunes where any water is frozen in a layer of ice atop high-pressure ice, with a thin H_2_-dominated atmosphere (65). **Hycean worlds**: Warmer than ice worlds, hycean worlds have a thin H_2_-rich atmosphere overlying an ocean of liquid water, which itself is above a layer of high-pressure ices. These planets have the potential to support habitable conditions (9). **Mini-Neptunes**: Sub-Neptunes with a deep H_2_-rich atmosphere followed by a mixed H_2_O-H_2_ supercritical layer below before reaching a layer of high-pressure ice and then a core (17, 65). **Steam worlds**: Water worlds with H_2_O dominated atmospheres. At equilibrium temperatures above ${\text{T}}_{\text{crit}}$, a high proportion of H_2_O exists in the atmosphere, with a supercritical mixed-envelope at higher pressures. **Supercritical mini-Neptunes**: These planets are hotter than ${\text{T}}_{\text{crit}}$, and contain interiors where H_2_ gas is highly soluble in supercritical H_2_O (46, 65). This mixed-envelope would lie over a layer of high-pressure ice above the core. **Legend**: The legend at the top shows the different compositions corresponding to the colors in the schematic, including the core, different phases of H_2_O and envelope composition; high-P ice refers to high-pressure ice. Note that the core has further distinct layers but we do not label them here. Sharp phase transitions are shown for ice-atmosphere and ocean–atmosphere transitions. Other phase transitions are marked by a gradient, as they may not be sharp phase transitions (83).

An emerging alternative explanation for muted spectral features in warmer sub-Neptunes is that their high temperatures increase the MMW which, in turn, decreases the spectral amplitudes. For example, as shown in Fig. 6, for volatile-rich sub-Neptunes with $T_{\mathrm{eq}}≳350$ K, the atmospheres are expected to be increasingly dominated by H_2_O, either as largely steam or a mixture of abundant H_2_O and H_2_. This would result in an increased MMW, leading to diminished spectral features. The ratio of H_2_O to H_2_ would depend on the amount of H_2_ present from formation and evolution. Consequently, smaller planets with lower H_2_ abundances are expected to have predominantly steam atmospheres, as observed for GJ 9827 d, whereas planets with inherently lower metallicities and low O/H ratios could have lower H_2_O abundances. Ultimately, the detection of atmospheric features will depend on both the presence of clouds/hazes and the MMW. Therefore, low-temperature sub-Neptunes with equilibrium temperatures below ∼400 to 500 K (40), are likely to be optimal targets for atmospheric characterization for given bulk properties.

### Mass–Metallicity Relation in the Sub-Neptune Regime.

The retrieved abundance constraints are beginning to allow comparative planetology in the sub-Neptune regime. The carbon abundance has served as a unique probe of planet formation in the solar system. The dominant form of carbon in the atmosphere of all solar system giant planets is CH_4_, which shows increasing abundance with decreasing planet mass (16). This trend has been used as an indicator of giant planet formation by core accretion (16). The CH_4_ abundances in the atmospheres of the ice giants Uranus and Neptune, with masses of 14.5 M_⊕_ and 17.2 M_⊕_, respectively, are at a few percent volume mixing ratio. An extrapolation of the solar system CH_4_ trend to lower masses predicts volume mixing ratios that are systematically higher for sub-Neptunes.

The abundances of cabon-bearing species derived for sub-Neptunes to date do not show strong evidence for increasing C/H with decreasing mass in this regime, i.e., below 10 M_⊕_, as shown in Fig. 5. Currently, abundance estimates of carbon-bearing molecules have been published for only two sub-Neptunes, K2-18 b (42) and TOI-270 d (45), where CH_4_ and CO_2_ have been detected. Accounting for the total carbon abundance by combining CH_4_ and CO_2_ still somewhat underpredicts the C/H ratio of TOI-270 d relative to the solar system trend, while K2-18 b is consistent. On the other hand, the retrieved carbon abundance for the exo-Neptune GJ 3470 b (53), which is close to Uranus and Neptune in mass, is consistent with the solar system trend; we note however that the detection significance of CO in this planet is relatively weak (53). Finally, no carbon-bearing molecule has been detected in GJ 9827 d, the smallest exoplanet in this sample (47). These initial estimates may indicate a carbon cliff or plateau in the mass–metallicity relation in the sub-Neptune regime. However, given the limited data available for only a few systems currently, it is too early to make a robust assessment of the trend. Nevertheless the ability to make such abundance measurements with JWST means that these initial indications can be tested with more precise abundance measurements for more planets in the future. Such measurements would help further refine the mass–metallicity relation and its implications for planetary formation in the sub-Neptune regime. On the other hand, Uranus, for which nitrogen abundance measurements are available, has nearly solar abundance value of NH_3_ (16), which is not detected in K2-18 b and TOI-270 d despite the favorable low temperatures where it would be expected for a deep atmosphere. These abundances potentially indicate a different internal structure and formation pathway for these temperate sub-Neptunes compared to the ice giants in the solar system.

### Cold Trap and Critical Temperature.

The atmospheric H_2_O abundance places important constraints on the atmospheric temperature structure and interior conditions. As shown in Fig. 5, the retrieved H_2_O abundance is found to increase with the equilibrium temperature of the planet, from a nondetection in the case of K2-18 b with *T*_eq_ below 300 K to over ∼30% for GJ 9827 d with a *T*_eq_ of 600 K. The observed trend is consistent with the presence of a cold trap in the atmosphere of K2-18 b whereby water is condensed at the tropopause leading to a dry stratosphere. On the other hand, for hotter sub-Neptunes the lack of a cold trap leads to observable water vapor in the atmosphere. Furthermore, a hot water rich interior will have H_2_O in supercritical phase in which H_2_ would be highly soluble, leading to a mixed envelope of H_2_ and H_2_O (65, 78). We refer to such planets as supercritical mini-Neptunes, as discussed below in *Classification of Volatile-Rich Sub-Neptune Interiors*; these have also been referred to as mixed envelope (65) or miscible envelope (46) sub-Neptunes. The high H_2_O mixing ratio observed for GJ 9827 d is consistent with this picture and can therefore be a supercritical mini-Neptune or a steam world, depending on the exact H_2_O vs H_2_ content. The observed trend also implies the presence of a critical temperature (T_crit_) dividing H_2_O-rich sub-Neptunes with a cold trap and low stratospheric H_2_O abundance and those without a cold trap and high H_2_O abundance. We note however that a subset of sub-Neptunes may be gas dwarfs, with much lower water abundances, which can result in such planets not following this trend.

While the value of T_crit_ is not yet known theoretically, an empirical estimate is possible based on the H_2_O abundance of TOI-270 d with *T*_eq_ of 352 K. Multiple estimates have been reported in the literature for the H_2_O abundance of TOI-270 d (45, 46). Both studies find the H_2_O abundance to be intermediate between K2-18 b and GJ 9827 d as shown in Fig. 5, albeit with significant uncertainty. The intermediate value would be consistent with the possibility of H_2_O being present in gas phase only on the dayside atmosphere with a cold trap on the nightside, consistent with expectations for a dark hycean world (9, 45).^†^ Based on this estimate, T_crit_ would be expected to be ≳350 K. On the other hand, if the true H_2_O abundance is closer to that of GJ 9827 d then T_crit_ would be expected to be below 350 K and the atmosphere would be more consistent with a mixed H_2_-H_2_O envelope as suggested by Benneke et al. (46). Therefore, a more precise H_2_O estimate of TOI-270 d could help establish the true value of T_crit_.

## Interiors and Surface Conditions

The atmospheric compositions are beginning to provide initial constraints on the internal structures and surface conditions of sub-Neptunes. One of the longest-standing questions in the field is about identifying which sub-Neptunes are gas dwarfs, mini-Neptunes, or water worlds. Atmospheric measurements are essential to break the internal structure degeneracy and to explore the diversity of possible internal structures.

### Degeneracies in Internal Structures.

In the absence of atmospheric measurements, a degenerate set of internal structures can usually explain the planetary mass and radius. For example, as shown in Fig. 1, the masses and radii for the three sub-Neptune targets K2-18 b, TOI-270 d, and GJ 9827 d all lie on the pure H_2_O curve. While their bulk properties can be explained by interiors composed entirely of H_2_O, such a prospect is unlikely based on planetary formation models, which require a nonnegligible rock component in planetary embryos. Conversely, the bulk parameters may be explained by varied mass fractions of a rocky core, H_2_O mantle, and H_2_-rich envelope, making it a degenerate problem (4).

As discussed above, the atmospheric compositions of several sub-Neptunes retrieved from JWST observations have revealed either H_2_-rich or H_2_O-rich atmospheres. While an H_2_O dominated atmosphere, e.g., of GJ 9827 d, makes the planet very likely a hot water world (a “steam world”), the presence of an H_2_-rich atmosphere, e.g., for K2-18 b or TOI-270 d, still allows for degeneracies in their internal structures. For example, the bulk parameters of K2-18 b could be explained by any the three sub-Neptune scenarios discussed above (6, 74), i.e., a gas dwarf, a mini-Neptune, or a hycean world. However, atmospheric constraints on of prominent molecules besides H_2_, such as CH_4_, CO, CO_2_, and NH_3_, are providing a powerful avenue to further resolve this degeneracy (18–20, 74). Such inferences are made using a combination of atmospheric and internal structure models for the different scenarios as discussed below.

### Atmospheric Signatures of Interiors and Surfaces.

Photochemical models have been used to predict expected atmospheric compositions for the three sub-Neptune scenarios with H_2_-rich atmospheres discussed above: a) mini-Neptunes, b) gas dwarfs, and c) hycean worlds. The relative abundances of chemical species in sub-Neptune atmospheres depend on various factors, including the atmospheric metallicity, internal temperature, incident radiation, dynamical processes, and the presence or lack of a surface (18–20, 42, 75, 76). Here, we briefly review the broad atmospheric predictions for each scenario, focusing on temperate to warm ($T_{\mathrm{eq}}\;∼\;$300 to 600 K) sub-Neptunes for which robust chemical detections have been made with JWST.

In temperate mini-Neptune atmospheres, with deep H_2_-rich atmospheres, H_2_O, CH_4_, and NH_3_ are expected to be the dominant molecules containing O, C, and N, respectively (17, 19, 75), as discussed in *Atmospheric Processes*. For warmer planets, CH_4_ and NH_3_ may be replaced by CO and N_2_, respectively, due to vertical mixing from deeper hotter regions. CO_2_ can also be abundant for high metallicity, and CO is expected to be more abundant than CO_2_, for solar-like elemental ratios. H_2_O may not be readily observable in very cool atmospheres due to condensation. The retrieved abundances of the warm exo-Neptune GJ 3470 b, as discussed in *Chemical Disequilibrium and Atmospheric Extent*, as well as the solar system ice giants are consistent with this composition. Mini-Neptunes are expected to follow the same abundance patterns as ice giants but with higher metallicities, if the mass–metallicity relation from the solar system were to be extrapolated. However, it is possible that several sub-Neptunes could also be gas dwarfs with rock dominated interiors and low volatile abundances in the atmospheres and hence not match this relation.

The expected atmospheric composition for gas dwarfs is similar to the mini-Neptune scenario, with the common aspect being their deep H_2_-rich atmospheres (76). For gas dwarfs with high enough temperatures and thick enough atmospheres the rocky surface below could be molten, leading to melt–atmosphere interactions that could affect the observed abundances, while still predicting CO > CO_2_ (21). An additional consideration is the possibility of a reduced mantle which can act as a sink for nitrogen (79, 80). However, a molten surface or N-depletion is not necessarily always feasible for temperate sub-Neptunes, and assuming so (81) can result in unphysical solutions (76). Another key difference is that atmospheric metallicities for gas dwarfs with just a rocky interior and an H_2_-rich envelope, would be expected to be lower than those of mini-Neptunes which are predicted to have high elemental abundances due to accreted ices during formation (13).

The expected atmospheric compositions for hycean planets represent a significant departure from both the mini-Neptune and gas dwarf scenarios. Given their thin H_2_-rich atmospheres their observable compositions are significantly affected by the presence of an ocean surface below which curtails the recycling of key molecules such as CH_4_ and NH_3_ from the deeper atmosphere which is in thermochemical equilibrium to the upper, observable, atmosphere where they can be photochemically destroyed (18–20, 74). The prominent molecules in such atmospheres are expected to be CO_2_, that is photochemical in origin or outgassed from the ocean, and/or CH_4_, that survives photochemistry or is biogenic. A key indicator of a hycean atmosphere is CO_2_ > CO and depletion of NH_3_ owing to both photochemical destruction and its high solubility in the ocean (18, 74, 82). While H_2_O may be depleted in temperate hycean atmospheres where a cold trap may be present (74), it could be observable in dark hycean atmospheres with warmer daysides (9). Finally, for an inhabited hycean world, the atmosphere may contain observable quantities of biogenic gases such as DMS, CS_2_, OCS, CH_3_Cl, and N_2_O (9).

### Classification of Volatile-Rich Sub-Neptune Interiors.

An emerging picture is that sub-Neptunes with significant volatile inventories (i.e., of H_2_ and H_2_O), including mini-Neptunes and hycean worlds, may represent a continuum in internal structures as a function of incident irradiation and atmospheric extent (46, 65, 84). A schematic of a classification in this two-dimensional space is shown in Fig. 6, with *T*_eq_ representing the irradiation and atmospheric extent representing the amount of H_2_ available which in turn affects the total atmospheric pressure. For a H_2_ dominated atmosphere the total atmospheric pressure (P_atm_) would be close to the partial pressure of H_2_ (PH_2_) with minor contributions from other gases. For a thin atmosphere, e.g., PH_2_ ∼ 1 bar, low temperatures (*T*_eq_ ≲ 250 K) can lead to an icy world with a dry H_2_-rich atmosphere over an icy surface. For intermediate temperatures, *T*_eq_ ∼ 250-T_crit_ K, the atmosphere may still be depleted in H_2_O due to a cold trap but the surface can be warm enough to sustain liquid water, resulting in a hycean world. At still higher temperatures, *T*_eq_ > T_crit_, a cold trap would no longer be possible, resulting in a steam world where H_2_O dominates the atmospheric composition, with supercritical H_2_O in the interior acting as a large sink for H_2_, given the high solubility of H_2_ in supercritical H_2_O (65, 78, 85). As discussed above, current empirical estimates suggest a T_crit_ ≳ 350 K.

For a given *T*_eq_, the surface temperature below the atmosphere increases with total pressure. For intermediate pressures, PH_2_ between approximately one and a few tens of bar, at low *T*_eq_ the atmosphere may still sustain a hycean world for a high enough albedo. For higher temperatures, the atmosphere would continue to be H_2_ dominated for *T*_eq_ < T_crit_ while a cold trap still persists. A cold trap in such a scenario, either throughout the atmosphere or only partially such as on the cooler night side, may still prevent substantial H_2_O in the atmosphere. However, the surface and interior would be in supercritical phase followed by high-pressure ice at depth, resulting in a mini-Neptune structure. For, *T*_eq_ > T_crit_, the lack of a cold trap would result in a high H_2_O abundance in the atmosphere and a supercritical mixed envelope of H_2_ and H_2_O (46, 65), which we refer to as a supercritical mini-Neptune. This state is equivalent to the mixed envelope (65) or miscible envelope sub-Neptune (46) scenario referred to in previous works. We use the term supercritical to distinguish this state from mixing that can occur in planetary interiors by other means, e.g., through dynamical processes. For higher PH_2_, a hycean world may no longer be possible, and the internal structure would be that of a mini-Neptune for *T*_eq_ < T_crit_ or a supercritical mini-Neptune otherwise. For supercritical mini-Neptunes, the amount of H_2_O in the atmosphere depends on the relative mass fractions of H_2_ and H_2_O available. Planets with lower H_2_O mass fractions in the interior, possible due to low ice accretion during formation, would be expected to have lower H_2_O abundances in the atmosphere.

### JWST Constraints on Sub-Neptune Interiors.

The retrieved atmospheric abundances provide initial constraints on the internal structures as discussed above. For K2-18 b, the abundances of CH_4_ and CO_2_ and nondetections/upper-limits of CO and NH_3_ are consistent with prior predictions for a hycean world (18, 20, 42). The constraint on the photospheric temperature and nondetection of water are also consistent with the presence of a cold trap expected at those temperatures (74). Recent studies investigating alternate scenarios highlight the challenges in explaining the retrieved abundances without a hycean scenario. Models of the mini-Neptune scenario with a deep H_2_-rich atmosphere (73, 86, 87) are unable to explain the CO/CO_2_, NH_3_, and/or H_2_O constraints (75). Other studies have highlighted the importance of self-consistent and physically plausible models. For example, ref. 88 considered a supercritical ocean to explain the CH_4_/CO_2_ ratio and CO nondetection without considering the effect of photochemistry or nitrogen chemistry. Similarly, another study considered the possibility of magma oceans to explain the NH_3_ depletion (81) while being inconsistent with the CO/CO_2_ constraints, planet mass and/or bulk density, and other factors (76).

The sub-Neptune TOI-270 d has very similar atmospheric features to K2-18 b (Fig. 4), with CO_2_ and CH_4_ detected, and also H_2_O, but not NH_3_ or CO. This indicates that TOI-270 d could be a dark hycean world, without a cold trap on the day side, but with one on the night side (9, 45). A separate analysis of the data detected the same molecules but with significantly higher abundances, by ∼1 dex, indicating a higher metallicity atmosphere (46). The abundances were used to infer a supercritical mini-Neptune with no liquid water surface. Both interpretations are likely plausible for the corresponding retrieved abundances, considering the dark hycean scenario is also expected to be supercritical on the dayside. However, more accurate observations and establishing the cause of the differences between the two analyses can help resolve the debate.

Finally, the hotter and smaller exoplanet, GJ 9827 d, with a high abundance of H_2_O in its atmosphere, is consistent with predictions for a steam world (47). The observed composition indicates an H_2_O-rich atmosphere over a mixed H_2_-H_2_O envelope, due to supercritical H_2_O, with a rocky mantle/core at depth. The source of the water could be primordial accretion or magma–atmosphere interactions (21, 22, 47). The bulk density of the planet precludes a predominantly rocky interior as shown in Fig. 1. Sulfur and carbon-bearing species, if present, may inform whether the inferred water stems from the accumulation of icy materials or from volatile-deficient formation with subsequent interior-atmosphere evolution (47, 86).

## Habitability and Biosignatures

Exoplanetary systems allow a wide range of possible environments that can exist within the habitable zone. Three types of temperate exoplanets have been considered to be conducive for habitable conditions: a) rocky worlds, b) ocean worlds, and c) hycean worlds. While rocky planets have traditionally been the primary focus in the search for habitability and biosignatures on exoplanets, temperate sub-Neptunes that can be ocean worlds or hycean worlds are emerging as promising avenues in recent years.

Habitable rocky worlds, which include temperate Earth-like planets and super-Earths, have densities consistent with interiors that are entirely rocky. Atmospheric observations of habitable rocky planets orbiting Sun-like (G dwarf) stars, which is the primary motivation for the Habitable Worlds Observatory (HWO) in the future (89), are beyond the capability of JWST. However, such planets around small nearby M dwarfs are accessible to JWST with significant investment of observing time. Recent JWST observations of several terrestrial-size planets in the TRAPPIST-1 system (90) have highlighted the significant challenges resulting from spectral contamination from the very active late M dwarf star as well as the planets potentially lacking significant atmospheres (62, 91, 92). An additional challenge is the small number of known transiting planets around nearby bright M dwarfs, though this could change with new planet detections, such as the recently discovered Gliese 12 b (93, 94).

Ocean worlds are temperate water-rich planets with ocean-covered surfaces and terrestrial-like atmospheres (8). Such planets can have a wide range of water mass fractions, including terrestrial-like rocky interiors but with more water than on Earth, e.g., 10 to 1,000× Earth oceans (95, 96), as well as planets with over 50% of their mass in water. While many known sub-Neptunes may be water worlds (15, 97) most are likely steam worlds that are too hot to be habitable. Recent JWST observations have identified a potential temperate ocean world, LHS 1140 b, inferred by its spectrum being inconsistent with a H_2_-rich atmosphere, indications of a high MMW atmosphere, and the bulk density being too low for an Earth-like interior (55).

Hycean worlds, as discussed above, are temperate water-rich planets with ocean covered surfaces and H_2_-rich atmospheres (9). Their lower densities, and hence larger radii, and larger atmospheric scale heights make them more conducive to detection and atmospheric characterization compared to rocky or ocean worlds of similar mass. The greenhouse warming due to the H_2_-rich atmosphere significantly expands the classical terrestrial HZ thereby increasing the available targets for atmospheric observations (9); over a dozen promising hycean candidates are known around nearby M dwarfs. As discussed in the above section, JWST observations and the retrieved atmospheric properties of the habitable zone sub-Neptune K2-18 b are consistent with prior predictions for a hycean world (42). Subsequent JWST observations of a second sub-Neptune TOI-270 d also indicate the possibility of a dark hycean world, albeit the retrieved properties are presently debated (45, 46).

Overall, hycean worlds present a promising avenue in the search for habitable conditions with JWST, both in terms of observability and the number of potential targets available. However, open questions remain about the interpretation of the atmospheric observations, as discussed above, and the potential of current hycean candidates for sustaining habitable conditions. When considering a planet’s climate, the Bond albedo and atmospheric extent determine whether a temperate sub-Neptune can be a hycean world. For K2-18b, an adequate albedo (e.g., ∼0.5 to 0.6) is required to maintain a liquid water surface (98, 99); zero-albedo models predict temperatures too hot to be habitable (98, 100, 101). While the observed transmission spectrum provides a 3σ evidence for clouds/hazes at the terminator (42), with the retrieved haze properties consistent with the required theoretical values (99), the dayside albedo is still unconstrained. However, considering the range of Bond albedos in the solar system planets with significant atmospheres is between ∼0.3 and 0.8, including 0.29 for Earth (102), 0.50 for Jupiter (103) and 0.76 for Venus (104), the required Bond albedo for K2-18 b may not be unusual (75). It is also presently unknown if the required thin/moderate atmosphere, with total pressure below ∼10 bar depending on the albedo, can be retained on K2-18 b given the susceptibility to atmospheric loss. Finally, while the current chemical abundances are most consistent with a hycean world scenario, it remains to be seen whether new atmospheric processes not considered previously might explain the observed abundances without the need for a hycean scenario. More precise observations and theoretical studies in the future could help answer these questions.

Recent studies have explored the nature and detectability of possible biosignatures on different types of habitable exoplanets with JWST. For terrestrial-like atmospheres, the detection of ozone (O_3_) on a TRAPPIST-1 planet would require several tens of transits, over hundreds of hours, with JWST (105). Habitable sub-Neptunes, such as hycean worlds, are more favorable for biosignature detectability due to their larger sizes and lighter atmospheres than rocky planets (9). Some of the prominent terrestrial biosignatures such as O_2_ or O_3_ are unlikely to be abundant in H_2_-rich atmospheres and others such as CH_4_ may have abiotic false positives. However, several of the secondary and less abundant biosignatures on Earth may be good biosignatures on hycean worlds. These include molecules such as dimethyl sulfide (DMS), CH_3_Cl, OCS, and CS_2_, some of which were also known to be present in the H_2_-rich atmosphere of the early Earth (106, 107). Theoretical studies have demonstrated that such biosignature molecules are both feasible in significant abundances and detectable with modest amounts of JWST time for super-Earths with H_2_-rich atmospheres (108) and hycean worlds orbiting nearby M dwarfs (9, 109). A very tentative inference of DMS was reported using recent JWST observations of K2-18 b (42). While it remains to be seen whether future observations confirm the finding, these initial observations demonstrate the potential of JWST to detect such molecules in sub-Neptune atmospheres.

## Summary and Future Outlook

For the past decade prior to JWST, the sub-Neptune regime had been a formidable frontier of exoplanet science. Within its first two years, JWST has already unveiled stunning glimpses of this exotic landscape with unprecedented detail. Robust chemical detections have been reported for several sub-Neptunes, starting with the habitable zone planet K2-18 b and followed by warmer planets TOI-270 d and GJ 9827 d. These results are already providing the insights into atmospheric processes and interiors/surface conditions, as well as population-level trends motivating a uniform classification scheme for volatile-rich sub-Neptune interiors. Finally, these insights have opened a new avenue in the search for life, through ocean worlds and hycean worlds.

These observations have taught important lessons both on sub-Neptune properties as well as their observability. First, the chemical detections across the sub-Neptunes observed with JWST suggest a high likelihood for water-rich interiors (42, 46, 47, 86). They also provide empirical evidence that temperate planets with H_2_-rich atmospheres are more readily observable than hotter planets with equivalent bulk properties due to the possibility of high H_2_O abundances and, hence, high MMW in the latter, e.g., for planets such as GJ 9827 d. Second, temperate sub-Neptunes below ∼400 K, such as K2-18 b and TOI-270 d, also seem to have less spectral attenuation due to clouds/hazes compared to their warmer counterparts. Third, atmospheric spectroscopy of sub-Neptunes orbiting active M dwarfs can be severely impacted by stellar heterogeneities and flares (55, 60). Planets orbiting mid-M dwarfs with low-moderate activity are more conducive for such observations. Finally, prominent molecules such as CH_4_, CO_2_, and H_2_O in temperate sub-Neptune atmospheres are within reach of JWST, often requiring minimal observing time for optimal targets. Furthermore, potential biomarker molecules are also detectable in promising sub-Neptune atmospheres with JWST, including species like DMS, CS_2_, CH_3_Cl, N_2_O, and OCS (9, 108, 109).

All the sub-Neptunes with significant atmospheric detections to date show signs of chemical disequilibrium. The inferred abundances also provide initial evidence for diverse internal structures, from steamy water worlds like GJ 9827 d (47) to possible hycean worlds like K2-18 b (42). It is important to note that these are still very early inferences and further observations and theoretical efforts are needed for more robust characterization of such planets. Nevertheless, these early insights are already motivating the atmospheric classification of volatile-rich sub-Neptunes as a function of their irradiation and bulk properties, spanning a continuum between hycean worlds, steamy worlds, mini-Neptunes, and supercritical mini-Neptunes. They also pave the way for constructing the mass–metallicity relation in the sub-Neptune regime with implications for planetary formation and evolution in this uncharted territory. Ultimately, however, the high sensitivity of JWST observations necessitate robust data reduction and analysis procedures, high-fidelity atmospheric retrieval frameworks and accurate self-consistent interior/atmospheric models for a comprehensive understanding of the sub-Neptune population.

The next steps on the sub-Neptune frontier with JWST are in two directions: wider observations of a broader range of targets enabling population-level studies, and deeper characterization of promising sub-Neptunes. Both approaches are essential to answer major open questions in this frontier: how do the atmospheric compositions of sub-Neptunes vary across the bulk properties between terrestrial planets and ice giants? How diverse are the internal structures of sub-Neptunes and what determines the transition between super-Earths and sub-Neptunes? Finally, what are the prospects for habitability and biosignatures in the sub-Neptune regime? The present work highlights the major advances that are happening in our understanding of sub-Neptunes with JWST. These insights, however, are but a glimpse of what vistas await in the JWST era. Further observations promise to solve longstanding mysteries about this exotic yet ubiquitous planetary class. This quest may even bring us face to face with one of humanity’s earliest and most primal questions: are we alone in the universe?

## Data Availability

There are no data underlying this work.

## Footnotes

^*^The habitable zone is considered to be the region around a star in which a planet can host liquid water on its surface (9, 43, 44).

^†^A dark hycean is where the liquid water ocean is present only on the night side of the planet (assuming the planet is tidally locked).
